# Uncovering gaps in personalised lung cancer care in Germany: a white spot analysis

**DOI:** 10.1186/s12885-025-15411-2

**Published:** 2025-12-12

**Authors:** Scarlett Berressem, Lars-Thorben Moos, Johanna Röder, Laura Harzheim, Nils Dreiack, Marlene Brodersen, Florian Kron, Robert Dengler, Hans Christian Reinhardt, Martin Utzig, Thomas Illmer, Jürgen Wolf, Anna Kron

**Affiliations:** 1 Department I of Internal Medicine, Medical Faculty, University of Cologne, University Hospital Cologne, Cologne, Germany; 2 Center for Integrated Oncology Aachen Bonn Cologne Duesseldorf (CIO ABCD), Cologne, Germany; 3 VITIS Healthcare Group, Cologne, Germany; 4 Federal Association of Outpatient Hematologists and Medical Oncologists (BNHO e. V.), Cologne, Germany; 5 National Network Genomic Medicine Lung Cancer, Cologne, Germany; 6 Federal Association for Outpatient Specialist Medical Care (BV-ASV), Munich-Grünwald, Germany; 7 Department of Hematology and Stem Cell Transplantation, Faculty of Medicine and University Hospital Essen, University of Duisburg-Essen, Essen, Germany; 8 German Cancer Society (DKG), Berlin, Germany

**Keywords:** White spot analysis, Comprehensive molecular diagnostics, Quality-assured clinical networks, Incidence-based evaluation, Personalised medicine in lung cancer, Inpatient real-world data

## Abstract

**Background:**

Lung cancer is among the most prevalent and deadly cancers, with around 57,000 new cases annually in Germany. Despite advances in targeted therapies, many patients still lack sufficient access to state-of-the-art comprehensive molecular diagnostics and personalised therapy throughout the disease course. This study identifies gaps in access to personalised lung cancer care in Germany. A primary objective was to analyse how many lung cancer patients are not captured by the established quality and network structures - namely, centres of the national Network Genomic Medicine (nNGM) and the lung cancer centres certified by the German Cancer Society (DKG) - to identify and ultimately close these gaps.

**Methods:**

Publicly available data were used from inpatient quality reports, the databases of nNGM and certified lung cancer centres (DKG-LZ), the outpatient specialist medical care programme (ASV) supplemented by incidence data from the German Centre for Cancer Registry Data (2022). Differences among nNGM, DKG-LZ and ASV were assessed, focusing on non-affiliated hospitals as proxies for putative lack of quality-assured molecular diagnostic access. Geographic mapping was used to visualise regional disparities.

**Results:**

The analysis included 993 hospitals with a total of 167,216 reimbursed lung cancer cases. 240 hospitals with 98,797 cases (59%) are affiliated with the nNGM. 108 hospitals with 78,591 cases (47%) are certified or cooperate with a DKG-LZ. ASV is provided at 152 hospitals or ASV partner hospitals (71,763 cases, 43%). nNGM and DKG jointly cover 276 hospitals and 114,472 cases (69%). Adding ASV, overall quality-affiliated coverage increases to 124,419 cases (74%). However, 668 hospitals with a total of 42,797 cases (26%) are not affiliated with any of these structures. Geographic analysis revealed regional disparities, notably in Northern Brandenburg, Lower Saxony, and Saxony-Anhalt characterised by high-incidence areas and non-affiliated hospitals managing large volumes of lung cancer cases.

**Conclusions:**

Significant disparities in access to quality-assured molecular diagnostics persist. Expanding nNGM and integrating high-volume hospitals could raise coverage to over 85%. Standardising molecular diagnostics and quality requirements across nNGM, DKG, and ASV could further optimise access to high-quality personalised lung cancer care in Germany and build a unified real-world evidence base for equitable, consistent care.

**Supplementary Information:**

The online version contains supplementary material available at 10.1186/s12885-025-15411-2.

## Background

With approximately 57,000 new cases annually, lung cancer is one of the three most common cancers in Germany. It also remains the leading cause of cancer-related mortality and accounts for the highest global healthcare costs [1, 2]. Even among non-smokers, lung cancer ranks as the seventh leading cause of death [3]. Due to the non-specific nature of early symptoms, more than 65% of lung cancer patients are diagnosed at an advanced stage, where curative surgery is no longer an option. However, over the last 15 years, a global decline in relative mortality rates has been observed. This trend is largely attributed to advancements in diagnostics and the development of targeted therapies, driven by a deeper understanding of molecular subtypes of lung cancer [1, 4].

A paradigmatic shift in lung cancer therapy occurred with the introduction of mutation-specific targeted drugs. Since 2009, when the EGFR-inhibitor gefitinib was approved in EGFR-mutated lung cancer in Europe, many such personalised therapies have been established and exist now for around one third of all lung cancer patients. However, these therapies require upfront comprehensive molecular diagnostics to identify specific driver mutations. The routine use of next-generation sequencing (NGS) from the initial diagnosis of lung cancer has been widely adopted nationwide by the national Network Genomic Medicine (nNGM) which currently comprises 29 specialised centres, delivering molecular diagnostics and therapy recommendations for more than 545 regional partners. An external evaluation demonstrated the significant benefit of the nNGM in terms of prolonged overall survival through a direct comparison with a non-nNGM group based on health insurance data [5]. Today, the nNGM serves as a key framework for molecular diagnostics and personalised treatment of lung cancer patients in Germany. The nNGM centres provide standardised NGS diagnostics at initial diagnosis and at each progression (re-biopsy) along with specific and up-to-date clinical information on in-label therapies, off-label treatment, and ongoing clinical trials. Additionally, they offer interdisciplinary clinical consultation, particularly through joint tumour boards, to ensure comprehensive and personalised treatment planning. Full reimbursement for NGS testing and consultations is provided through selective contracts under section 140a Social Code Book (SGB) V with the majority of the statutory health insurances. These contracts establish and enforce specific high-quality standards to ensure optimal patient care and achieve nationwide harmonization of requirements for all nNGM healthcare providers. Intersectoral regional partners, including 255 hospitals and 290 private practices (as of 31 October 2024) ensure accessible, close-to-home patient care while adhering to the highest standards of medical innovations. The nNGM provides care for approximately 17,000 patients annually, covering around 60% of the estimated incidence of advanced-stage lung cancer patients [6].

In addition to the nNGM, certified lung cancer centres (DKG-LZ) of the German Cancer Society (Deutsche Krebsgesellschaft (DKG)) serve as quality-assured structures providing lung cancer care for approximately 43% of initial lung cancer diagnoses annually [7]. In order to receive or renew official certification, the DKG-LZ must demonstrate high-quality through regular external audits, which specifically assess adherence to strictly defined and publicly accessible structural and performance criteria [8]. The DKG certification is valid for 3 years, requires an annual surveillance audit, and is not tied to any additional or specific reimbursement regulations. The requirements for molecular diagnostics in the certified lung cancer centres are based on the strong recommendations and quality indicators of the German S3-guideline and require molecular diagnostics for relevant mutations in NSCLC stage IV and EGFR and ALK analysis in anatomical resected stage IB-IIIA. An independent comparative cohort study (WiZen) based on claims data and population-based data from cancer registries demonstrated generally positive outcomes for patients treated at various DKG-certified centres, particularly regarding high-quality surgical procedures performed at these centres. For lung cancer, the WiZen study demonstrated significant outcome benefits for patients treated at DKG-LZ, based on cancer registry data [9].

Furthermore, outpatient specialist medical care (Ambulante Spezialfachärztliche Versorgung (ASV)) is a specific financial model designed for outpatient services in complex and severe diseases, including lung cancer. The ASV is structured around multidisciplinary teams providing comprehensive diagnostics, including NGS, and treatment. Quality assurance is based on the requirements of the Joint Federal Committee (Gemeinsamer Bundesausschuss (G-BA)) guideline on ASV, which mandates, for example, a minimum of 70 patients treated annually [10, 11]. Proof of quality is usually provided through documentation and reporting obligations. However, there are currently no specific requirements for molecular diagnostics rates or related quality indicators, and as a result, no published case numbers are available. Nevertheless, it is reasonable to assume that adherence to specific quality standards is required to ensure full reimbursement. Currently, outcome data related to ASV are not available.

Despite the established clinical networks such as nNGM, certifications from the DKG and specific quality-adjusted reimbursement options like ASV, the available data reveal devastating gaps in lung cancer care, as not all lung cancer patients in Germany receive comprehensive molecular diagnostics and personalised treatment [12]. The reasons for missing molecular diagnostics are multifactorial, ranging from structural and financial barriers to a lack of overall quality controls and regional differences in the availability and utilization of testing services [13, 14]. The actual numbers remain unclear due to a lack of systematic investigations, further compounded by inconsistent or non-uniform documentation.

Given the current situation, the aim of this study is to systematically identify gaps in lung cancer care particularly in state-of-the-art molecular diagnostics during the entire course of the disease (so-called ´white spots”) in Germany. Specifically, the study will analyse geographical differences in the availability of qualified molecular diagnostics and access to clinical networks (nNGM) and other quality-assured or fully reimbursed structures such as DKG-LZ and ASV. Since lung cancer patients treated within the nNGM have been shown to experience improved overall survival [5] the overarching goal of this study is to evaluate the potential for maximizing nationwide integration into the nNGM. The analysis focuses on the inpatient sector due to the centralised availability of data.

## Methods

A white spot analysis, in the meaning of the above specified understanding of the terminology, was conducted to illustrate regional differences in lung cancer care. This method is a practical tool for assessing market potential in business administration and has been previously applied to healthcare and personalised medicine [15]. For this purpose, publicly available data from various lung cancer networks and hospitals were collected and supplemented with incidence data 2022 from the national Centre for Cancer Registry Data, aggregated at the district code level. Case numbers for hospitals were extracted from the G-BA reference database, which includes published hospital quality reports for 2022, using the ViSyMe software. Hospitals that reported at least five reimbursed lung cancer cases in 2022 and remained operational as of the survey cut-off date as of October 31 2024, were included in the analysis. Case definitions followed the criteria outlined in the quality reports, meaning that patients with multiple inpatient stays were counted as separate cases each time [16].

Lung cancer networks or care structures that adhere to defined quality criteria and undergo external review were classified as quality-assured. These included, in descending order of quality assurance regarding molecular diagnostics: (A) nNGM, (B) DKG-LZ, and (C) hospitals participating in the ASV for ´Tumours of the Lung and Thorax´. Other potential networks were excluded due to a lack of publicly available data.

The locations of nNGM centres and regional partners, as well as DKG-LZ including specialised departments (DKG-LZ cooperation partners) such as haematology/oncology, pulmonology, thoracic surgery, pathology, and clinical trial units - were mapped and compared with the hospitals included in the survey as of October 31, 2024. Additionally, ASV teams, categorised into lead teams and core team/cooperating specialists, were extracted from the ASV Service Centre website and assigned to hospitals. Outpatient institutions were generally excluded, except for molecular pathology facilities relevant to molecular diagnostics.

Hospitals that could not be assigned to any of the identified quality-assured structures (nNGM, DKG-LZ or ASV) but reported at least 30 lung cancer cases per year were contacted via an online questionnaire. Priority was given to hospitals with the highest case numbers based on the 2022 quality reports. The questionnaire focused on collaboration with partner institutions and participation in molecular diagnostic networks. It was titled “Survey of Inpatient Care Structures for Patients with Lung Cancer in Germany” and was developed using Empirio, a web-based platform for creating and distributing scientific questionnaires. The survey was distributed via email to selected contacts. Prior to distribution, a systematic search for relevant email addresses was conducted. The primary target group included medical directors (or their administrative offices) of departments of internal medicine or haematology, as well as oncology centre coordinators and representatives of quality management. The questionnaire was initially sent on 7 April 2024, with follow-up reminders on 29 April 2024 and 13 May 2024. The complete set of survey questions used in the online questionnaire is available in the Supplementary Material (Supplementary File 1). Furthermore, an extensive online search was conducted to identify pathological institutes and private practices with potential network affiliations and cooperation agreements.

To identify and visualize ´white spots” in lung cancer care, the open-source geographic information system QGIS was used. Location data for hospitals and regional partners, along with incidence figures, were imported and mapped. The estimated number of affected patients was calculated by multiplying the relative incidence at the municipal level by the number of inhabitants in the corresponding postal code area, assuming incidence to be uniformly distributed within each municipality. This approach also enabled a more precise representation of urban centres, as incidence data at the postal code level were not available. Distance measurements were performed using the MMQGIS module in QGIS.

## Results

### Inclusion of hospitals and case number coverage by networks

**Fig. 1:**
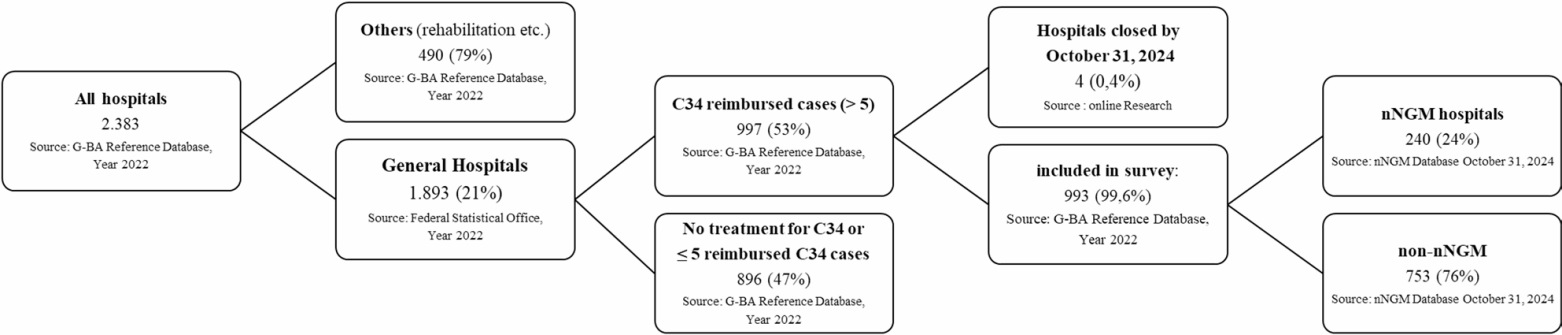
Consort diagram, shows the number of hospitals and cases included in the survey and the coverage by the nNGM

**Fig. 2:**
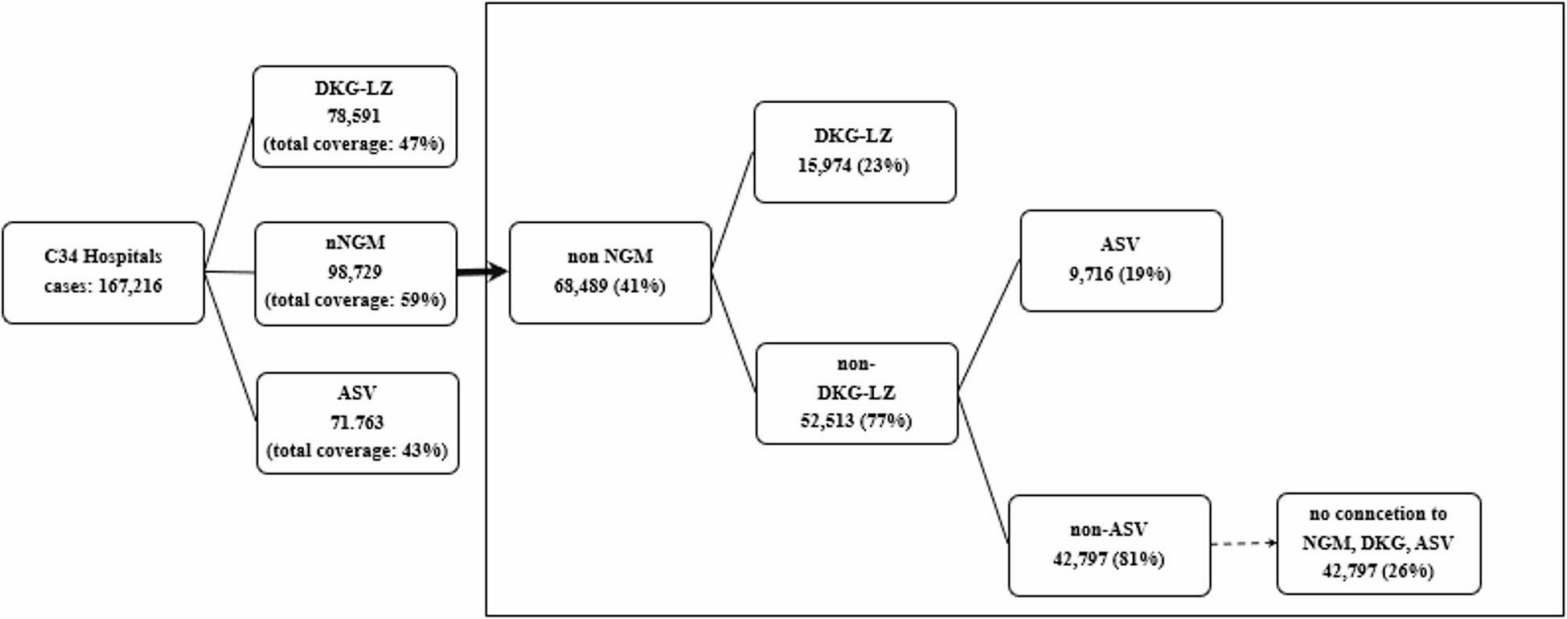
Consort diagram: Figure of coverage of hospitals with C34 cases by nNGM, nNGM + DKG-LZ, nNGM + DKG-LZ + ASV/lead teams and hospitals without connection

A total of 993 hospitals were identified, accounting for a total of 167,216 reimbursed lung cancer cases (ICD-10 diagnosis code C34) in 2022 (Figs. 1 and 2). Among these, 240 hospitals (24%) were affiliated with the nNGM (A), covering 98,729 cases, which represents 59% of all cases. In total, 753 hospitals (76%) were not affiliated with the nNGM, managing 68,487 cases (41%) (Fig. 1).

**Fig. 3:**
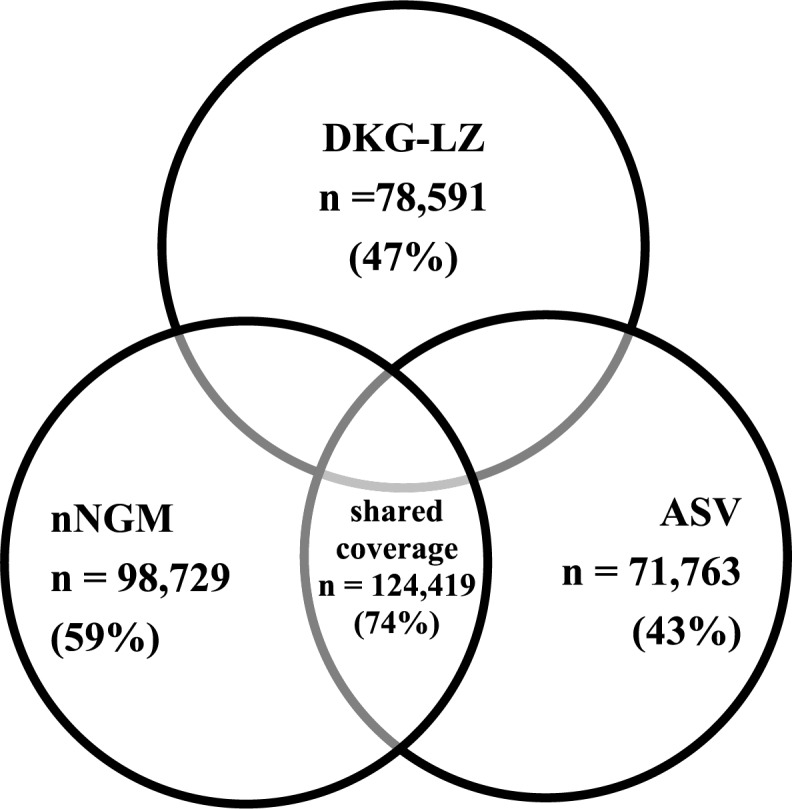
Single and shared coverage by reimbursed case numbers and connection to nNGM, DKG and ASV

DKG-LZ covered 78,591 cases (47%) and ASV 71,763 (43%). The shared coverage of nNGM, DKG-LZ and ASV was 74% (Fig. 3). The highest number of lung cancer cases in non-nNGM hospitals was observed in the cities of Chemnitz, Nuremberg, and Münster. Nevertheless, patients in these cities have access to quality assured molecular diagnostics via DKG-LZ: among the non-nNGM hospitals, 36 hospitals (5%) were associated with the DKG-LZ (B), accounting for 15,974 reimbursed lung cancer cases (23%). Hospitals not affiliated with the nNGM or a DKG-LZ account for a total of 52,513 C34 cases. Within this cohort, 49 hospitals participated in the ASV, covering 9716 cases. A total of 668 hospitals, with a case volume of 42,797, were not affiliated with the nNGM, the DKG-certified lung cancer centres, or the ASV (A + B + C). This corresponds to 26% of all lung cancer cases billed in Germany that were considered in this study. See Fig. 2 for further details as well as Fig. 3 to see the shared coverage of reimbursed cases of the three quality-assured network structures.

Table 1 illustrates the coverage of lung cancer cases by the respective quality-assured structures (A-C). The numbers are calculated individually, regardless of multiple counting, where applicable.

**Table 1:** Connection of the hospitals included in the survey to the various network structures

	Number of hospitals	Number of C34 cases 2022	Share of cases
Included in survey	993	167,216	-
nNGM Centre	29	25,312	15%
nNGM regional partner hospital	211	73,417	44%
nNGM Total	240	98,729	59%
DKG Lung Cancer Centre (DKG-LZ)	89	71,556	43%
DKG LZ cooperation partner	98 (19*)	74,304 (7,035*)	44% (4%)
DKG LZ Total	108	78,591	47%
ASV (lead team)	114	63,786	38%
ASV (core/cooperation specialists)	63 (38**)	19,736 (7,977**)	12% (5%**)
ASV Total	152	71,763	43%
nNGM + DKG	276	114,703	69%
nNGM + ASV	303	117,472	70%
nNGM + DKG + ASV	325	124,419	74%

* excluding hospitals that also have a certified lung cancer centre and were therefore already counted under ´DKG-LZ`

** excluding hospitals that also serve as ASV lead team hospitals and were therefore already counted under ´ASV (lead team)”

**Fig. 4:**
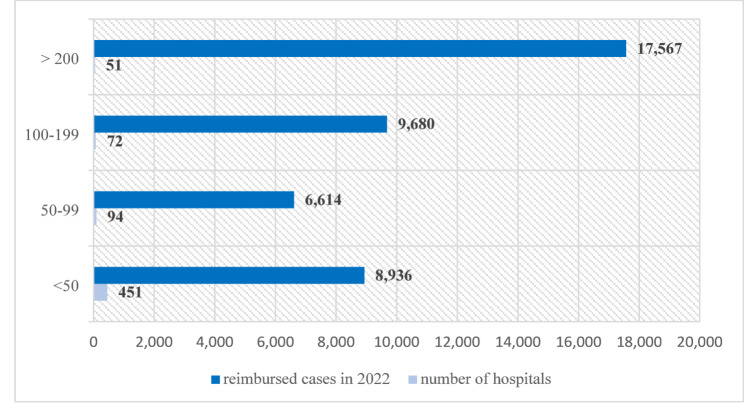
Hospitals without connection to nNGM, DKG-LZ or ASV according to number of reimbursed cases

668 hospitals not affiliated with the respective quality-assured structures (A-C) were categorised into four groups based on their reimbursed lung cancer cases (Fig. 4). A total of 51 hospitals (8%) with over 200 lung cancer cases accounted for 10% (*n* = 17,567) of the total case number in 2022. This corresponds to 41% of all reimbursed lung cancer cases at hospitals not affiliated with any of the quality-assured structures (A-C).

Additionally, 72 hospitals (11%) with 100–199 lung cancer cases accounted for 23%, 84 hospitals (14%) with 50–99 lung cancer cases covered 15% and 451 hospitals (68%) with fewer than 50 lung cancer cases covered 21% of all reimbursed lung cancer cases outside quality-assured structures.

### White spot analysis

**Fig. 5:**
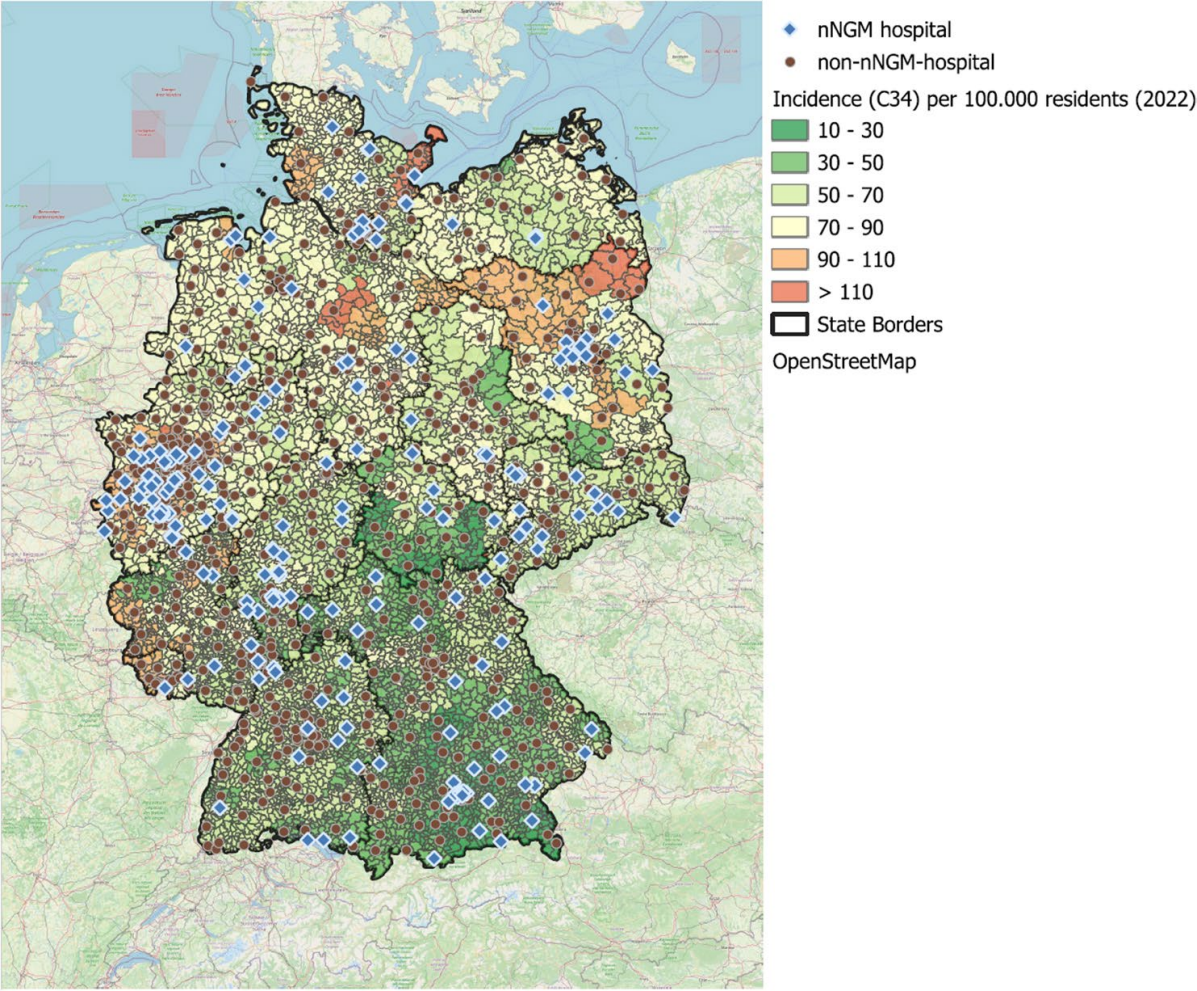
Map of Germany showing hospitals either associated with nNGM (as nNGM centre or contracted regional network partner) or not associated with nNGM

Figure 5 illustrates the distribution of nNGM-affiliated hospitals and non-nNGM hospitals in relation to the incidence of lung cancer (C34) at the municipal level.

**Fig. 6:**
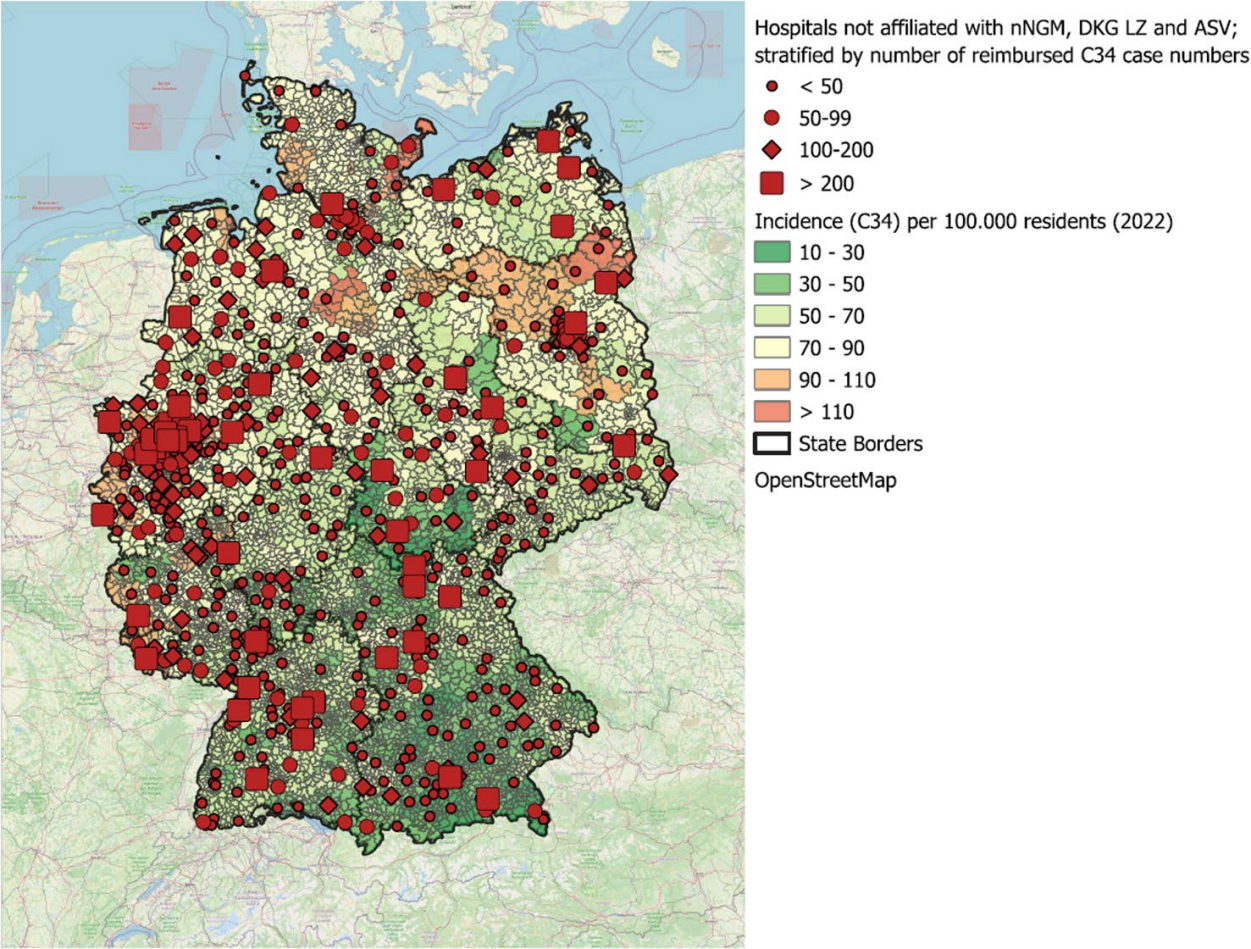
Map of Germany showing hospitals without affiliation to structures A-C, stratified by number of reimbursed lung cancer cases

Figure 6 illustrates the locations of hospitals not affiliated with any of the quality-assured structures (non-nNGM, non-DKG, non-ASV) stratified by reimbursed C34 case numbers, overlaid with the lung cancer incidence from 2022 at the municipal level (as published in 2023). Additionally, a version of Fig. 6 without stratification is provided in the Supplementary Material (Supplementary Fig. 1).

**Fig. 7:**
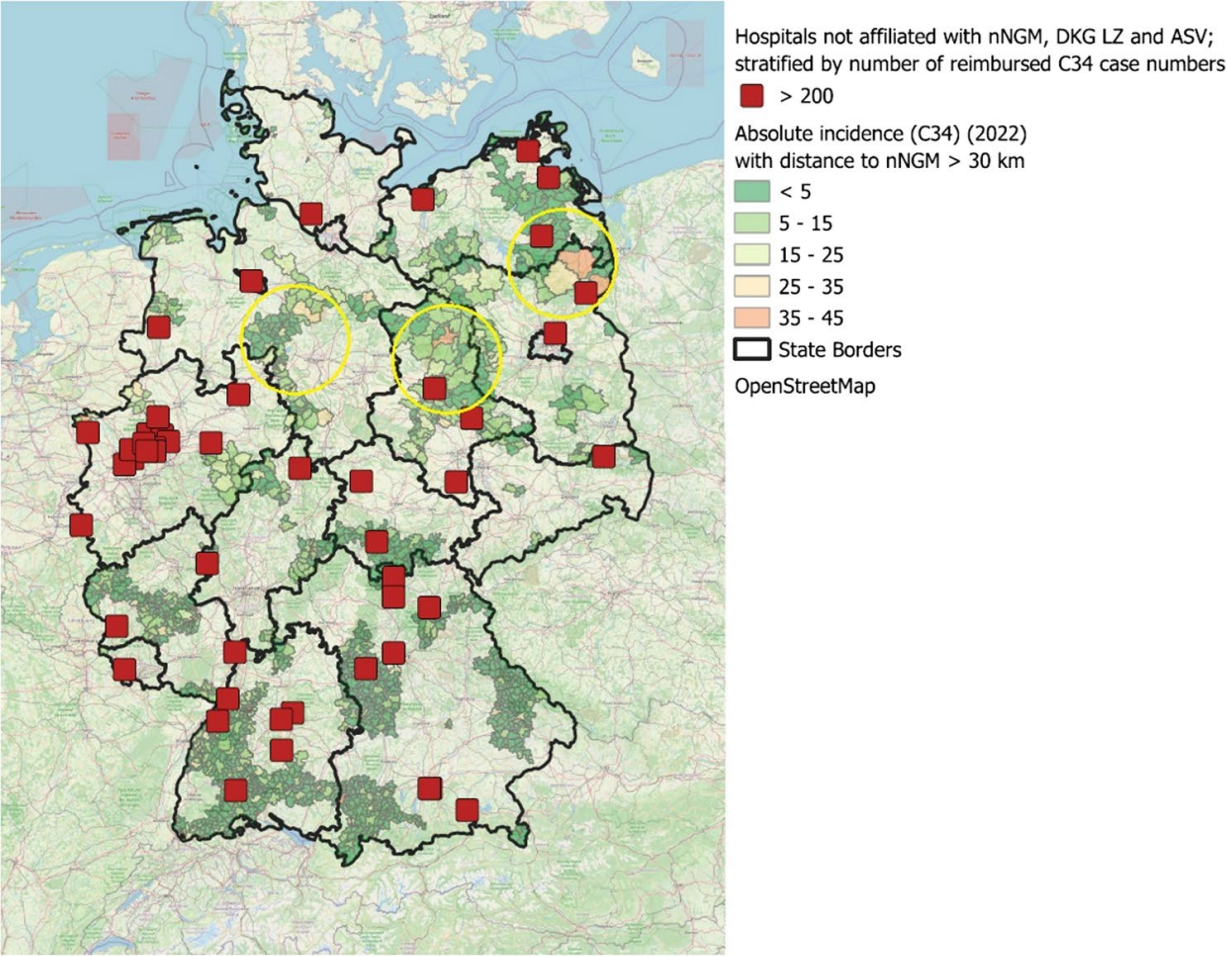
Map of Germany showing areas with long distances to the nearest nNGM-affiliated hospital, with hospitals stratified by number of reimbursed lung cancer cases

Figure 7 highlights areas where the nearest nNGM-affiliated hospital is more than 30 km away. The colour intensity varies according to lung cancer incidence levels in these regions. Areas without additional coloration indicate that a nNGM centre or a regional partner hospital is accessible within a 30 km radius. Additionally, the map highlights hospitals that are not affiliated with any of the defined quality-assured structures but reported at least 200 lung cancer cases in 2022 (red rectangles). Within the map of Germany, the three regions with the highest incidence rates of lung cancer (2022) - while simultaneously lacking proximity to an nNGM partner hospital - are circled. These regions are located in northern Brandenburg, central and south-eastern parts of Lower Saxony, and Saxony-Anhalt. The map is also provided in the Supplementary Material without the overlay of circles and hospital locations, to allow clearer regional interpretation (Supplementary Fig. 2).

### ASV pathology partners outside of nNGM

**Fig. 8:**
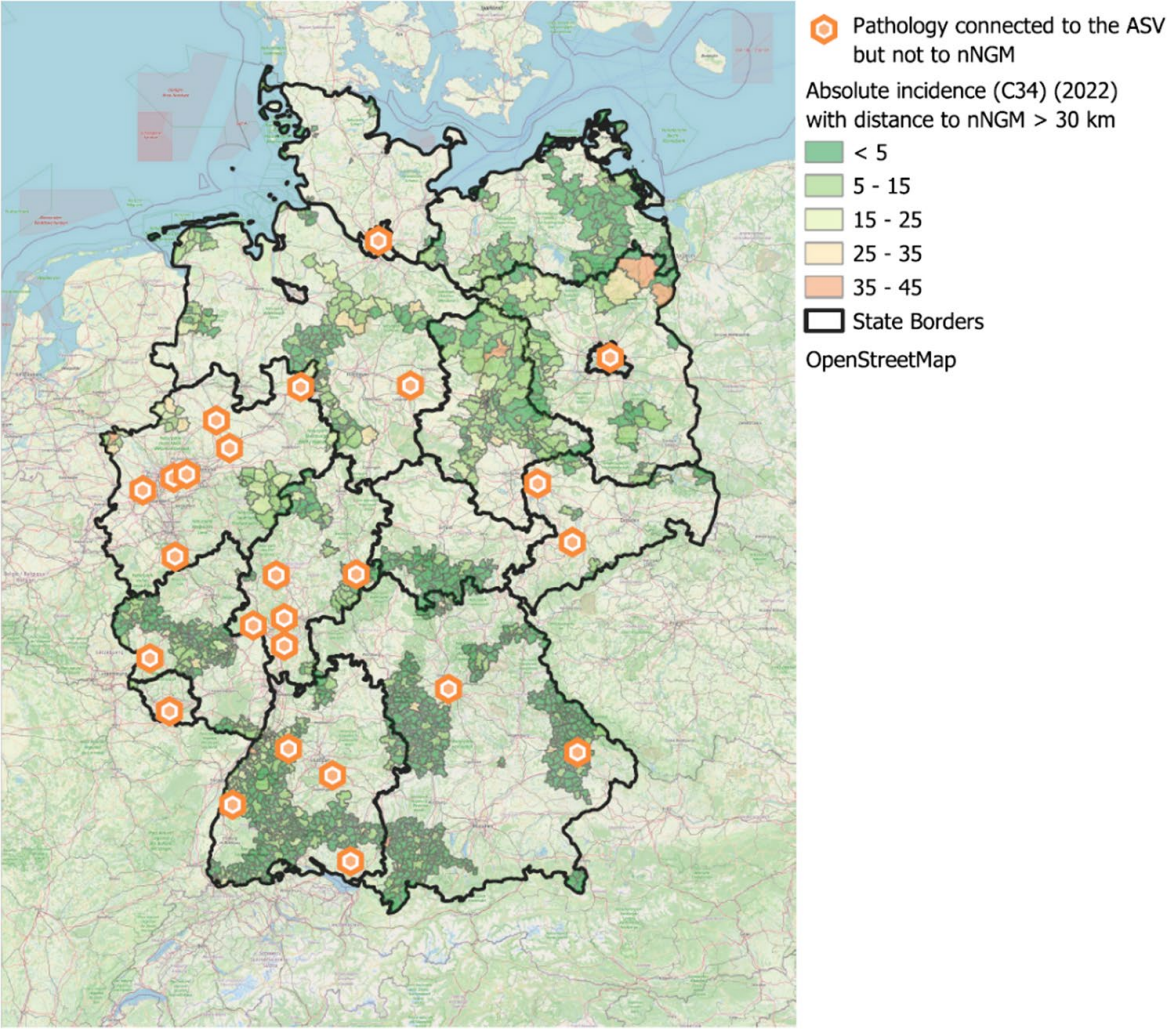
Map of Germany showing pathology institutes connected to ASV but not affiliated with nNGM. Highlighted areas indicate regions where the nearest nNGM-affiliated hospital is located more than 30 km away

In total, 42 hospitals participating in ASV did not cooperate with the nNGM, with approximately 60% located in North Rhine-Westphalia or Baden-Württemberg. These hospitals, in turn, collaborated with 25 pathology institutes outside the nNGM, the majority of which were situated in North Rhine-Westphalia, Baden-Württemberg, and Hesse. Figure 8 illustrates the pathology institutes within the ASV that were not connected to the nNGM. Areas where the nearest nNGM-affiliated hospital was at least 30 km away are highlighted in colour. Institutes within the ASV that are not connected to the nNGM.

### Additional questionnaire for hospitals

After identifying hospitals not affiliated with quality-assured structures (*n* = 51), those with at least 30 lung cancer cases in 2022 (*n* = 317) were contacted via digital questionnaire. Due to the limited availability of open-source information, the survey aimed to assess their potential providers of molecular diagnostics for lung cancer patients. However, with a response rate of 6.25%, the results are not representative.

## Discussion

This study aimed to provide a comprehensive overview of the current lung cancer care land-scape in Germany focusing on comprehensive molecular diagnostics as the cornerstone of personalised lung cancer therapy. Therefore, access to then NGM, DKG-LZ and/or ASV was evaluated in order to detect potential ´white spots” in lung cancer care. This study aimed to provide a comprehensive overview of the current lung cancer care landscape in Germany focusing on comprehensive molecular diagnostics as the cornerstone of personalised lung cancer therapy. Therefore, access to then NGM, DKG-LZ and/or ASV was evaluated in order to detect potential ´white spots” in lung cancer care.

The nNGM has been established as a model for personalised lung cancer care in Germany. Based on its proven impact on patient outcomes – including a significant survival benefit demonstrated through independent evaluations using both nNGM and statutory health insurance data [5] – the network sets a national benchmark for high-quality molecular diagnostics and complex treatment in lung cancer. All patients enrolled in the nNGM receive comprehensive molecular profiling at the time of initial diagnosis and at disease progression, accompanied by structured interdisciplinary case discussions. Most patients are subsequently treated close to home within cross-sectoral care structures - including hospitals and outpatient practices - ensuring personalised, high-quality care throughout the entire disease course. Thus, the core strength of the nNGM lies not in molecular diagnostics alone, but in the integrated, multidisciplinary management of the entire patient journey. This is made possible by close collaboration across specialties and care sectors. Key elements include the interdisciplinary interpretation of molecular results, the development of evidence-based treatment recommendations, and the longitudinal evaluation of real-world-data and patients follow-up, all of which contribute to a consistent, high-quality standard of lung cancer care nationwide.

Other long-established quality-assured programmes, such as the DKG-certified lung cancer centres, primarily focus on structural and process quality, adhering to predefined standards but not specifically prioritising comprehensive molecular diagnostics or long-term patient follow-up. Moreover, DKG certification does not provide additional funding for any medical services. At the same time, a strong partnership between nNGM and DKG institutions is both feasible and beneficial, fostering synergies in lung cancer care and enhancing patient access to high-quality molecular diagnostics.

This study also included ASV as a quality-relevant structure. However, ASV is primary de-fined as a specific trans sectoral care model for complex outpatient care where hospital and practicing physicians participate. The quality standards in ASV are subject todifferent requirements than those in the nNGM or DKG, and are therefore not equivalent nor harmonised. In particular, quality parameters related to access to personalised lung cancer therapy — such as the scope and quality of molecular diagnostics, access to clinical trials, and treatment outcomes — are not systematically collected. Nevertheless, comprehensive molecular diagnostics is fully reimbursed under the ASV, thereby enhancing patient access to these essential services. nNGM centres, along with their regional partners, and DKG-LZ as well as their cooperation partners also participate in ASV. Therefore, this study assumes an overlap in service provision, particularly concerning molecular diagnostics and its quality requirements within the ASV framework.

Thus, in contrast to the DKG certification and ASV, the nNGM not only ensures quality assurance but also offers full reimbursement through a flat-rate model for both inpatient and outpatient molecular diagnostics and consultations.

According to this study, then NGM covers approximately 60% of all inpatient lung cancer cases in Germany (Fig. 2). These results align with the network’s published data and under scores its central role in lung cancer care. Similarly, for the DKG-LZ, the detected cover-age of 43%, increasing to 47% when including cooperating partners, aligns with the official reports [16]. Considering both nNGM and DKG-LZ, these networks together would cover approximately 69% of all inpatient lung cancer cases. Involving the ASV centres, even though the quality requirements here are not comparable to those of the nNGM the total coverage could increase to approximately 74% of cases within quality-assured or at least quality-related and reimbursed structures.

Consequently, more than aquarter of lung cancer cases (*n* = 42,797 in 2022) in Germany are treated in hospitals outside of the mentioned above quality-assured structures and networks. This also raises concerns about the extent, reliability and evaluation of comprehensive molecular diagnostics in these institutions. This applies to 668 hospitals in total, with only 51 of these institutions accounting for nearly half of all cases not affiliated with nNGM, DKG-LZ or ASV. The majority of these hospitals are located in North Rhine-Westphalia, likely due to the high total population in this federal state. At the same time, every hospital in Germany, regardless of its affiliation with DKGor ASV has the opportunity to join the nNGM as a partner, thereby ensuring secure access to harmonised molecular diagnostics and network services for all patients.

The geographical ´White Spot” analysis reveals significant regional differences in access to molecular diagnostics, particularly in northern Brandenburg, Lower Saxony, and Saxony-Anhalt, where accessibility to nNGM centres or their network partners may be limited due to longer distances (Fig. 6). A targeted expansion of molecular pathology services in these regions, for example by strengthening network collaborations, should be prioritised to im-prove patients access to high-quality diagnostics. Hospitals with high case volumes (*n* > 200 cases per year) represent particularly promising strategic points for enhancing lung cancer care and expanding access to molecular diagnostics. Expanding the nNGM to include these hospitals could increase the overall coverage to over 85% of inpatient lung cancer cases, thereby substantially enhancing the quality of care (Fig. 3). This expansion would not only provide direct benefits for patients treated in these hospitals but also create positive spill over effects on regional care structures, further improving access to high-quality molecular diagnostics and personalised treatment.

An additional factor supporting the focus on high-volume hospitals is the Hospital Care Act, which came into force on January 1, 2025 [17]. This legislation introduces new regulations and restrictions on hospital reimbursement for lung cancer surgeries, making it likely that the number of hospitals treating lung cancer will decrease, while the number of cases man-aged in larger centres will continue to rise.

It is important to note that not all patients in the identified underserved regions necessarily completely lack access to molecular diagnostics. As shown in Fig. 8, some of these regions host molecular pathology facilities that provide diagnostics for ASV hospitals, particularly in Eastern Hesse, Bavaria, and Western Baden-Wüürttemberg. However, these molecular pathology institutes currently do not cooperate with the nNGM. Potential collaborations with these institutions could enhance quality standards, ensure a more harmonised approach to molecular diagnostics, and improve accessibility for surrounding areas. 

The results of this study highlight significant regional disparities in access to quality-assured molecular diagnostics for lung cancer patients as the basis for personalised treatment and provide concrete starting points for improving regional care structures, based on the white spot analysis. Fifteen years after the approval of the first targeted therapy for lung cancer and given the well-documented survival benefits of targeted treatments [18, 19], the current level of inpatient integration into quality-assured structures providing molecular diagnostics remains insufficient. Previous studies have already demonstrated a substantial under utilization of molecular diagnostics in lung cancer patients in Germany [11]. The current analysis not only underscores this structural gap, affecting approximately 25% of all lung cancer patients, but also, for the first time, identifies specific areas for targeted action.

When comparing with similar analyses from other European countries, it becomes apparent that few data are available demonstrating nationwide coverage of molecular testing for lung cancer. In France, a prospective national cohort study evaluated testing rates in approximately 9000 lung cancer patients treated at non-academic public hospitals [20]. The study re-ported that nearly 88% of eligible patients received molecular testing. This high rate is likely attributable to the early establishment of a national network of 28 Molecular Genetic Centres for Cancer (MGCC), initiated by the French National Cancer Institute (INCa) as early as 2007, providing a robust and centralised testing infrastructure. Other countries have also launched structured initiatives to promote access to molecular diagnostics. In the United Kingdom, for example, the NHS Genomic Medicine Service (GMS) was introduced to standardise and integrate genomic testing into routine care. The GMS is built around seven regional Genomic Laboratory Hubs (GLH), which coordinate molecular testing across defined catchment areas [21]. However, real-world data on implementation and national coverage have not yet been published, making a comprehensive assessment of its effectiveness currently unfeasible. In Germany, the establishment of clinical care networks is challenged by the federal structure with varying administrative practices and even more by the strict separation between the inpatient and outpatient care sectors, which has both structural and economic implications. This multi-level fragmentation complicates patient follow-up along uniform care pathways and limits the ability to conduct comprehensive evaluation. This study constitutes the first comprehensive assessment of cross-regional and cross-administrative lung cancer care structures in the German healthcare system. To ensure adequate and equitable access to molecular diagnostics, a systematic expansion of quality-assured networks within the inpatient and outpatient sectors is urgently required. Beyond nationwide integration of hospitals into these networks, it would also be beneficial to extend the harmonised quality criteria defined by nNGM, for which, for the first time also on an international level, a survival benefit was demonstrated, to other institutions within the DKG and ASV. This would help standardise molecular diagnostic quality, ensuring consistency and accessibility across all oncology care settings. In particular, DKG certification asa DKG-LZ should adhere to the same molecular diagnostic requirements as those established within the nNGM. Additionally, harmonizing certification standards across both networks would be highly beneficial, making them more transparent, accessible, and understandable for both healthcare providers and patients. This would visibly ensure participation in the nNGM within the context of DKG-LZ certification, guaranteeing standardised access to high-quality molecular diagnostics. The same quality standards as those in the nNGM should also apply to the ASV, provided that molecular diagnostics is fully funded within these structures. To obtain a more comprehensive picture of intersectoral care, future analyses should expand the scope to include outpatient care structures and relevant professional organisations.

Despite the robustness of the present findings, certain limitations must be acknowledged. A complete depiction of the real-world care landscape was not entirely possible, as detailed information on additional collaborations in molecular pathology remains scarce. Some in-formation on the ASV Service Centre website is not publicly accessible. Therefore, it can be assumed that the actual number of ASV teams is slightly higher. Despite extensive research and direct contact with hospitals, only limited data on non-publicly documented networks could be obtained. In order to gain further insights into the testing practices by hospitals not affiliated with the three quality-assured frameworks, an additional online survey was con-ducted. However, the response rate was disappointingly low at just 6%, limiting the representativeness of the findings. The reasons for this low rate remain speculative. One possible explanation is the difficulty in identifying appropriate contacts within the targeted hospitals. The majority of these hospitals do not have an informed coordinator in place. As a result, it is conceivable that no staff member felt sufficiently informed to complete the questionnaire. Further barriers may include heavy clinical workloads, data protection concerns, and the absence of incentives to participate. A substantially higher response rate might have enabled a more detailed understanding of care structures not yet captured by existing frameworks. It is also possible that some hospitals collaborate with external pathology institutes to provide molecular testing, and that they cooperate with regional(outpatient) network partners of the nNGM as part of continued care, but without formal contracting. Based on the findings of this study, these hospitals can now be proactively approached by the nNGM to explore and potentially establish formal collaborations. An nNGM affiliation would help harmonize care structures, ensure quality and data access, and thereby improve knowledge transfer, ultimately leading to better patient outcomes.

While hospital-related data, such as DKG certification, is increasingly available through the Federal Hospital Atlas [22], there is no comparable centralized registry for outpatient hemato-oncologists or molecular pathology facilities in Germany. As a result, this analysis primarily focuses on the hospitals and inpatient sector. Furthermore, it should be noted that molecular testing is not required for all patients with lung cancer; however, as non-small cell lung cancer (NSCLC) accounts for approximately 85–90% of all cases and testing is recommended from stage IB onwards, the majority of patients are subject to molecular diagnostic evaluation under current guidelines [23]. It should also be noted that both there lative and absolute incidence in areas with low population can be distorted and are not completely robust.

## Conclusions

Certain regions in Germany like northern Brandenburg, Lower Saxony, and Saxony-Anhalt would benefit from an expansion of quality-assured molecular pathological care, providing a critical foundation for equitable access to personalised treatment for lung cancer patients. Integrating these 51 additional hospitals into nNGM, could potentially increase overall coverage by at least 10%. Future studies should also incorporate outpatient care structures to provide a comprehensive representation of intersectoral care pathways and further optimize access to high-quality molecular diagnostics and personalised treatment to improve patient’s outcome. Our goal is to achieve nationwide coverage within the nNGM structures, and with full integration and quality-assured harmonization of all existing lung cancer care structures, such as those of the DKG and ASV.

## Supplementary Information

Supplementary Material 1.

Supplementary Material 2.

Supplementary Material 3.

## Data Availability

All data analysed (nNGM, ASV, DKG, G-BA quality reports) are publicly accessible, except for the incidence data, and are additionally available upon reasonable request from the corresponding author. The incidence data can only be obtained from the Centre for Cancer Registry Data.

## Abbreviations

ASV: Ambulante Spezialfachärztliche Versorgung (outpatient specialist medical care)
DKG: Deutsche Krebsgesellschaft (German Cancer Society)
DKG-LZ: Deutsche Krebsgesellschaft Lungenkrebszentrum (German Cancer Society-certified lung cancer centres)
G-BA: Gemeinsamer Bundesaussschuss (Joint Federal Committee)
GLH: Genomic Laboratory Hubs
GMS: NHS Genomic Medicine Service
INCa: French National Cancer Institute
MGCC: 28 Molecular Genetic Centres for Cancer
NGS: Next Generation Sequencing
nNGM: nationales Netzwerk Genomische Medizin (national Network Genomic Medicince)
SGB: Sozialgesetzbuch (Social Code Book)
