# Supplementary figures and images for: Uncovering gaps in personalised lung cancer care in Germany: a white spot analysis
Source: BMC Cancer. 2025 Dec 12;26:89. doi: 10.1186/s12885-025-15411-2 (PMC12822196; doi:10.1186/s12885-025-15411-2)

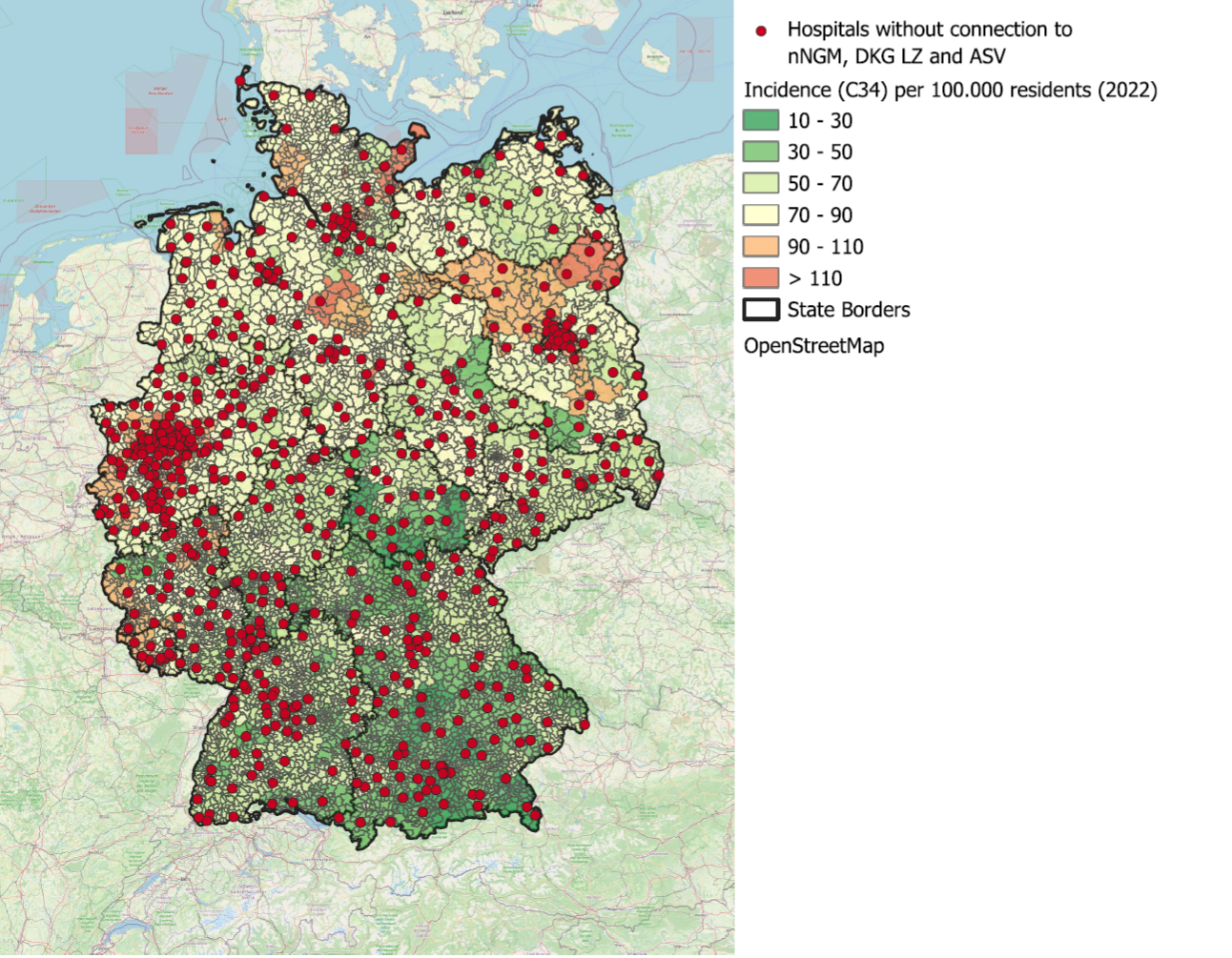

Supplement: Supplementary file 1 — Supplementary Material 1. [file 12885_2025_15411_MOESM1_ESM.png]

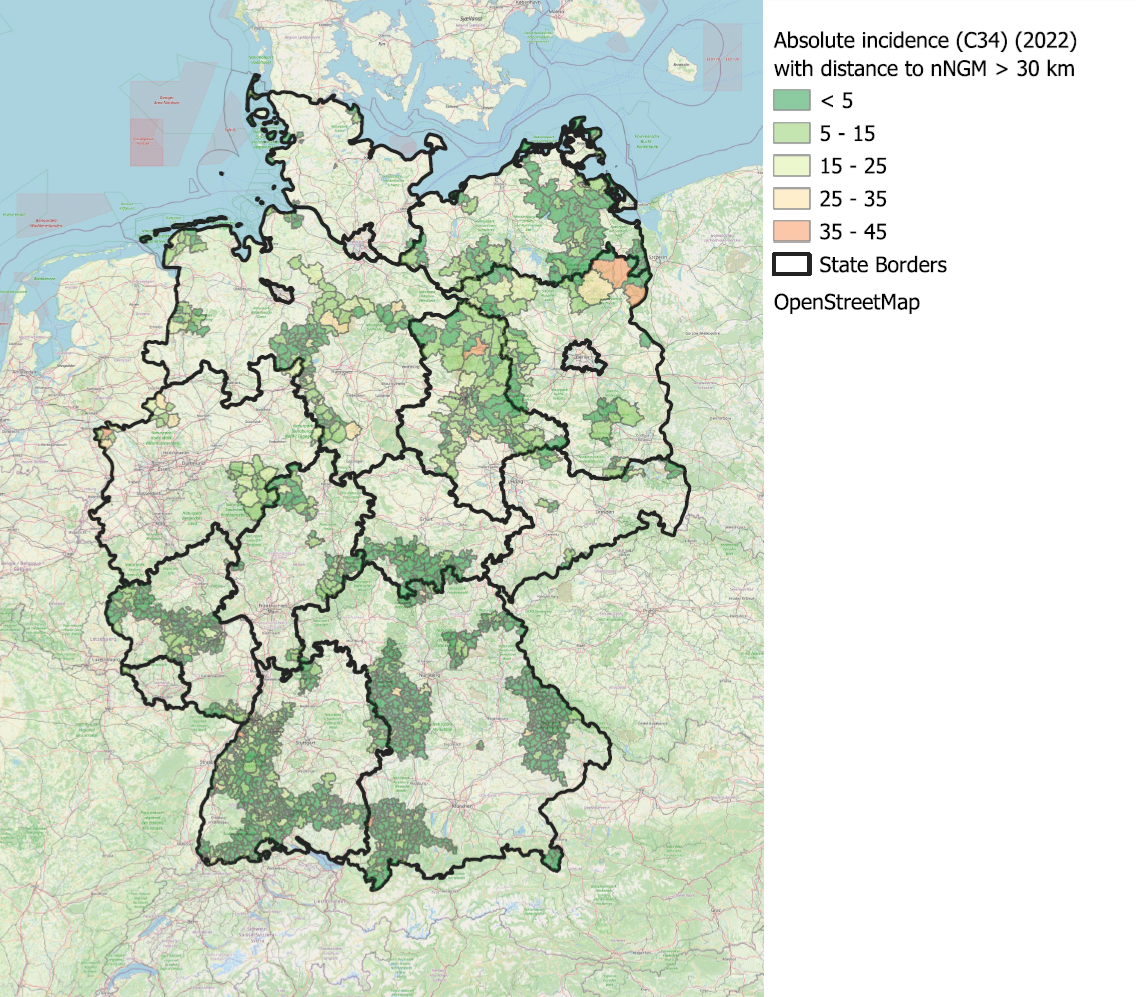

Supplement: Supplementary file 2 — Supplementary Material 2. [file 12885_2025_15411_MOESM2_ESM.png]
