# Supplementary material for: Uncovering gaps in personalised lung cancer care in Germany: a white spot analysis
Source: BMC Cancer. 2025 Dec 12;26:89. doi: 10.1186/s12885-025-15411-2 (PMC12822196; doi:10.1186/s12885-025-15411-2)
Supplement: Supplementary file 3 — Supplementary Material 3. [file 12885_2025_15411_MOESM3_ESM.pdf]

**Survey of Inpatient Care Structures for Patients with Lung Cancer in Germany**

**Please provide general information about your site (Hospital name, postcode, city):**

---

---

**Does your hospital have a department of haematology/oncology?**
☐ Yes ☐ No

---

**Does your site operate a medical care centre (MVZ) specialising in pulmonary medicine?**
☐ Yes ☐ No

---

**Does your hospital participate in outpatient specialised medical care (ASV)?**
☐ Yes ☐ No

---

**Do you perform molecular pathological diagnostics in lung cancer patients?**
☐ Yes ☐ No

*If yes: in which laboratory or institute is the molecular pathological testing conducted?*
(in-house or name of cooperating partner):

---

---

**What type of molecular pathological diagnostics is performed?** *(please tick one)*
☐ A: Single gene testing
☐ B: NGS – small panel
☐ C: NGS – large panel

---

**Do you collaborate with the national Network for Genomic Medicine (nNGM)?**
☐ Yes ☐ No

*If yes: which nNGM centre or location do you work with?*
Name of centre: ________________________

---

**Do you cooperate with a lung cancer centre certified by the German Cancer Society (DKG), e.g. as a partner institution?**

☐ Yes ☐ No

Name of centre: ______________________

---

**Do you conduct tumour boards in which lung cancer patients are discussed?**

☐ Yes ☐ No

*If yes: what types of tumour boards are conducted at your institution? (multiple answers possible)*

☐ Organ-specific tumour boards

☐ Molecular tumour boards

---

**Are you affiliated with any other existing network structure (apart from DKG-certified lung cancer centres or nNGM)?**

☐ Yes ☐ No

*If yes: which network/partner (lung cancer) are you affiliated with?*
Name of network: ______________________

---

**Would you be interested in joining a certified lung cancer centre or the national Network for Genomic Medicine (nNGM)?**

☐ Yes ☐ No
